# Isolation and genetic characterization of a novel Kevo orbivirus and a strain of Mobuck virus from Ochlerotatus communis mosquitoes in Finland

**DOI:** 10.1099/jgv.0.002101

**Published:** 2025-06-06

**Authors:** Maija T. Suvanto, Phuoc T. Truong Nguyen, Hanna Vauhkonen, Viktor Olander, Ruut Joensuu, C. Lorna Culverwell, Katariina Kaansalo, Jussi Hepojoki, Olli Vapalahti, Essi M. Korhonen, Teemu Smura, Eili Huhtamo

**Affiliations:** 1 Department of Virology, Faculty of Medicine, University of Helsinki, Helsinki, Finland; 2 Department of Veterinary Biosciences, Faculty of Veterinary Medicine, University of Helsinki, Helsinki, Finland; 3 Department of Geosciences and Geography, Faculty of Science, University of Helsinki, Helsinki, Finland; 4 Lammi Biological Station, Faculty of Biological and Environmental Sciences, University of Helsinki, Helsinki, Finland; 5 Vetsuisse Faculty, University of Zurich, Zurich, Switzerland; 6 HUS Diagnostic Center, Virology and Immunology, Helsinki University Hospital, Helsinki, Finland

**Keywords:** Finland, Kevo orbivirus, Mobuck virus, mosquito-borne virus, *Ochlerotatus communis*, orbivirus

## Abstract

The genus *Orbivirus* (*Reovirales*, *Sedoreoviridae*) comprises several globally important vector-borne animal viruses, such as *Culicoides-*borne bluetongue virus, African horse sickness virus and epizootic haemorrhagic disease virus (EHDV). Orbiviruses that are associated with mosquitoes are a diverse group including established mosquito-borne animal pathogens such as the Peruvian horse sickness virus and phylogenetically related less well-characterized viruses isolated mostly from mammals. Although reported from various geographic regions, these viruses have not previously been detected in northern Europe. Using next-generation sequencing and bioinformatic tools, we identified two orbivirus strains from virus isolation trials of Finnish *Ochlerotatus communis* specimens performed in mosquito C6/36 cells. Phylogenetic analysis of the obtained sequence data suggested one of the isolates to represent a strain of Mobuck virus (MBV), previously described in the USA from a diseased white-tailed deer coinfected with EHDV. The second isolate, which originated from Kevo in northern Finland, was found divergent from all publicly available orbivirus sequences and was tentatively named as Kevo orbivirus (KEVOOV). Further studies are required to investigate the potential animal disease associations of the newly detected orbiviruses KEVOOV and MBV in Finland and possibly wider in Northern Europe.

## Data summary

The viral sequences determined in this study are available at the NCBI GenBank under accession numbers PQ1657789-798 (KEVOOV) and PQ505671-2 and PQ165799-806 (MBV Ilomantsi strain). Supplementary material is available with the online version of this article, available through Figshare at https://doi.org/10.6084/m9.figshare.28760873 [1].

## Introduction

Orbiviruses (*Sedoreoviridae*, *Reovirales*) are widely distributed and globally important vector-borne animal viruses that are transmitted by various haematophagous arthropods, such as *Culicoides* biting midges, phlebotomine sandflies, mosquitoes and ticks [2,3]. The type species of the genus is the *Culicoides*-transmitted bluetongue virus (BTV) [3]. Orbiviruses have a triple capsid virion structure that shelters a 10-segmented dsRNA genome [3]. The genomic segments are numbered from largest to smallest, and seven of the genomic segments encode the structural proteins (VP1–VP7) and four encode the nonstructural proteins (NS1–NS5). Only segments 9 and 10 encode multiple ORFs [3].

Orbiviruses can infect a broad range of vertebrates, including ruminants, birds and rodents [2,4]. Some are zoonotic, such as the sandfly-borne Changuinola virus in Central and South America, and Asia, and the tick-borne Kemerovo virus in Russia, which have been reported to cause severe disease in humans [2,5, 6]. *Culicoides*-borne BTV, African horse sickness virus (AHSV) and epizootic haemorrhagic disease virus (EHDV) are economically significant and capable of causing large-scale outbreaks of severe, even fatal, animal diseases in domestic and wild ruminants, including equids [7,9]. Orbiviruses associated with mosquito vectors are a less characterized, diverse group of viruses that have been isolated from vertebrate hosts. The Peruvian horse sickness virus (PHSV)-related viruses form a phylogenetic group that includes viruses known to be mosquito borne, such as PHSV, isolated from Peru and Brazil [10,11] and Yunnan orbivirus (YUOV) isolated from China, Indonesia, Peru, Japan, the USA, and the Philippines [12,17]. In addition, this group also includes viruses isolated from animals for which the mosquito-vector association has not been defined, such as Cervidae Health Research Initiative (CHeRI) orbiviruses 1–3.4 [4,18], Mobuck virus (MBV) [19,20] and Lobuck virus (LBV) from the USA [21]; Elsey virus (ELSV) and Middle Point orbivirus (MPOV) from Australia [22]; Rioja virus from Peru [10]; and Yonaguni orbivirus (YONOV) [23,24] and Guangxi orbivirus (GXOV) from China and Japan [15,25]. In this study, we isolated and characterized the first two mosquito-associated orbiviruses detected in northern Europe, from mosquitoes collected in northern and eastern Finland.

## Methods

**Fig. 1.**
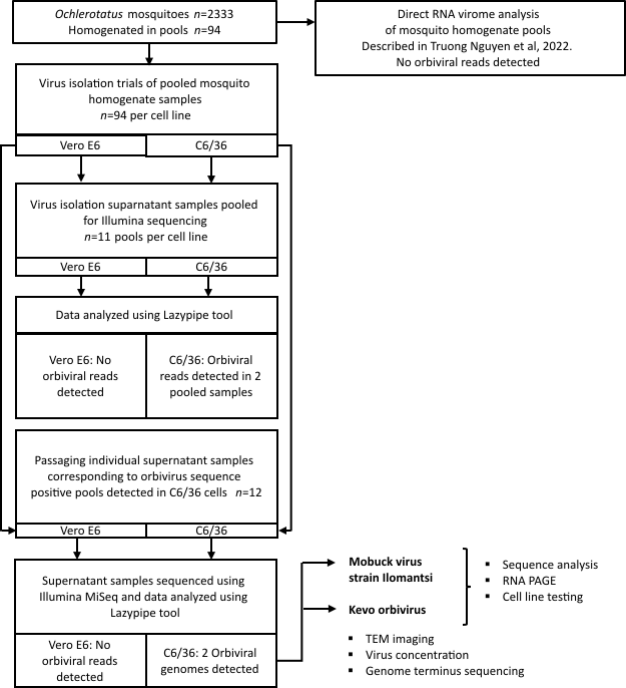
Flowchart of the study.

The flowchart of the study is described in Fig. 1.

### Virus isolation

The collection, identification, homogenization and pooling criteria of the 2,333 *Ochlerotatus* specimens that were used in this study are fully described in an earlier study [26]. In the present study, those 94 mosquito homogenate pools of 9 commonly encountered *Ochlerotatus* species from Finland were subjected to virus isolation trials in cultured mosquito and mammalian cells.

African green monkey kidney Vero E6 (ATCC CRL-1586) and C6/36 (ATCC CRL-1660) cells were maintained at 37 °C, 5% CO_2_ and at room temperature (RT) (~22 °C), respectively. Prior to inoculation, confluent T25 cm^2^ cell flasks were rinsed twice with PBS+antibiotics [a mixed solution containing penicillin 10,000 U ml^−1^ and streptomycin 10,000 U ml^−1^ (Lonza)]. A 450 µl aliquot of mosquito homogenate was taken, and 25 µl of antibiotics was then added. To inoculate the Vero E6 cells, 200 µl of this homogenate-antibiotic mix was pipetted directly onto the cell monolayer at RT. The flasks were then gently rocked by hand every 15 min for 1 h. After an hour, Minimum Essential Medium (MEM) (Sigma-Aldrich, USA) supplemented with 2% FBS, l-glutamine (Gibco) and penicillin-streptomycin was added to each flask, and the cells were transferred to, and maintained at, 37 °C 5% CO_2_. The remaining 250 µl of mosquito homogenate was filtered through a 0.45 µm Millex PES syringe filter (Merck) directly onto the C6/36 cells. To remove any remaining homogenate from the filter, 200 µl of Dulbecco’s PBS+0.2% BSA was then also filtered into the flasks. Filtering was performed to avoid bacterial and fungal contaminants that are often a problem in mosquito cell virus isolations of mosquito homogenates. The C6/36 cells were maintained at RT in Leibovitz’s L-15 medium (Gibco) supplemented with 2% FBS (Gibco) and antibiotics. Both cell lines were checked daily for cytopathic effects (CPEs), and cells and supernatants were harvested either when CPEs first appeared or on day 14 post-infection and then stored at −80 °C prior to further analyses.

### Pooling of virus isolation trial supernatants for initial sequencing

Supernatant samples originating from 94 virus isolation trials in C6/36 and Vero E6 cell samples were first combined into 11 superpools per cell line for initial Illumina MiSeq sequencing. A total of 100 µl of each individual virus isolation supernatant sample was combined and mixed, resulting in superpools that varied in their total volume between 200 and 1,500 µl, depending on how many individual culture supernatants were included (Table S4, available in the online Supplementary Material).

### Virus passaging

Based on the superpool sequencing results, superpool 5 was processed further. The superpool 5 was comprised of 12 initial virus isolation trial samples, which were individually passaged on clean Vero E6 and C6/36 cells. Even when viral reads were only detected from C6/36 cell isolation trials, the corresponding Vero E6 culture was also processed in a similar manner.

The passaging was performed on freshly confluent cells in T25 cm^2^ flasks, which were inoculated with 100 µl of the initial virus isolation trial supernatant sample for 1 h. The inoculum was then removed, and the cells were rinsed twice with PBS to remove any possible inoculum carryover, before adding fresh media of either MEM or L-15 (cell line dependent) with added antibiotics and 2% FCS. The inoculated cells were then incubated for 1 week and split as follows: the inoculated cells were detached and mixed with a small amount of clean cells and transferred along with the media from the inoculum bottle into a new 75 cm^2^ cell culture bottle. Finally, fresh media supplemented with 5% FCS were added. Cells were then observed for CPE and harvested when CPE was observed, or on day 14 after splitting. Cells and supernatants were stored at −80 °C prior to further analyses. This passaging step was conducted to identify which/how many of the individual pools contained virus and to confirm virus growth in cell culture. In the initial virus isolation trials, the inoculum mosquito homogenate or homogenate filtrates were not removed, and therefore, carryover of viral RNA from the homogenate was possible in the samples of the initial sequencing round.

### Sequencing and annotation of genomes using Illumina MiSeq and Lazypipe pipeline

A total of 200 µl of starting material (either pooled or individual cell culture supernatant sample) (Table S4) was used for RNA extraction using TRIzol reagent (Invitrogen, USA) with added glycogen, according to the manufacturer’s instructions, and dissolved in 25 µl of ddH_2_O.

A total of 17 µl of each RNA sample was treated with 1 µl DNase I (Thermo Fisher Scientific, USA) with 2 µl of reaction buffer for 30 min at 37 °C. The reaction was then stopped by adding 1 µl of EDTA. Library synthesis and amplification was then conducted according to a modified WTA2 (Complete Whole Transcriptome Amplification Kit) protocol with one-fifth of the volume of the original protocol (Sigma-Aldrich). After amplification, the DNA was purified using SPRIselect DNA beads (Beckman Coulter, USA), and libraries were prepared using Nextera DNA Flex Library Prep Kit (Illumina, USA). Sequencing libraries were then purified with SPRIselect DNA beads and sequenced using the Illumina MiSeq v3 (600 cycles) sequencing kit.

The resulting viral reads were pre-processed with fastp [27], assembled with MEGAHIT [28] and annotated using SANSparallel [29] implemented in Lazypipe pipeline [30], which first assembles all reads into contigs, annotates the reads based on the contig to which they were re-mapped and then calculates them per virus.

### Kevo orbivirus concentration for terminal nt sequencing

For obtaining a concentrated RNA sample for genome terminal sequencing, Kevo orbivirus (KEVOOV) was concentrated from infected C6/36 cell culture supernatants. A total volume of 90 ml of virus containing supernatant was harvested on day 14 post-infection and centrifuged at 3,000 ***g*** for 15 min, and 90 ml of clarified supernatant sample was then filtered through a 0.22 µm syringe-driven filter prior to loading on top of a 25% sucrose cushion for ultracentrifugation at 28,000 ***g*** at +4 °C for 2 h. The virus pellet was resuspended by pipetting it in 380 µl of sterile PBS. A total of 200 µl of concentrated virus was used for RNA extraction using TRIzol reagent (Invitrogen) with added glycogen according to the manufacturer’s instructions and used for terminal nt sequencing.

### Sequencing the terminal nt of KEVOOV segments using MinION

The sequencing of the terminal regions of the genomic segments was carried out using full-Length Amplification of cDNAs (FLAC) as described by Maan *et al.* [31] and Potgieter *et al.* [32] with slight modifications and long-read Oxford Nanopore Technologies (ONT, UK) MinION sequencing. A 5′ phosphorylated hairpin oligonucleotide (PC3-T7 5′-pGGATCCCGGGAATTCGGTAATACGACTCACTATATTTTTATAGTGAGTCGTATTA-OH-3′) was ligated to the 3′ ends of the dsRNA genome using T4 RNA ligase (Thermo Fisher Scientific) in a total volume of 20 µl using the following conditions: 1 µl of dsRNA (~1,500 ng µl^−1^, quantified with Nanodrop, Thermo Fisher Scientific), 1 µl of PC3-T7 (100 pmol µl^−1^) oligonucleotide (Metabion), 2 µl of 10× T4 ligation buffer, 2 µl (24% w/v) PEG 6000 (Thermo Fisher Scientific), 1 µl T4 RNA ligase (Thermo Fisher Scientific) and 13 µl H_2_O in +4 °C for 24 h. The ligated RNA/DNA heteroduplexes were purified using RNAClean XP Beads (Beckman Coulter) and dissolved in 20 µl of ddH_2_O.

The first strand cDNA was synthesized using Maxima RT reverse transcriptase (Thermo Fisher Scientific) utilizing the hairpin oligonucleotide’s 3′ end as a primer. After that, 5 µl of the purified ligation reaction, 1 µl deoxyribonucleotide triphosphates (dNTPs) and 4 µl H_2_O were denatured in 10 µl reaction volume for 2 min at 95 °C and cooled on ice. Four microlitres of 5× RT buffer, 0.5 µl of RiboLock RNAse inhibitor, 1 µl Maxima reverse transcriptase and ddH_2_O were added to the denatured RNA for a total volume of 20 µl. The mixture was incubated at 50 °C for 30 min and 80 °C for 5 min.

A total of 5 µl cDNA was used for subsequent PCR amplification using 12.5 pmol of amplification primer (PC2 5′-CCGAATTCCCGGGATCC-3′) and Q5 Hot Start High-Fidelity 2X Master Mix (New England Biolabs) in a 25 µl reaction volume. The PCR programme consisted of a 30-s initial denaturation at 98 °C, 35 cycles of 15 s at 95 °C and 5 min at 63 °C. The PCR products were purified using 1X SPRISelect beads and dissolved in 20 ul of ddH_2_O and quantified using Qubit 4 fluorometer (Thermo Fisher Scientific). Segment amplification was confirmed by agarose gel electrophoresis.

The sequencing was carried out using MinION Mk1C sequencer and R9.4.1 flow cells (ONT). The sequencing library was prepared using 250 ng of purified PCR product and ligation sequencing kit (SQK-LSK110, ONT), according to the manufacturer’s protocols. The library was quantified using a Qubit fluorometer, and 42.4 ng was used in the subsequent MinION run. The run was carried out overnight (~20 h) according to the manufacturer’s protocols using a QC cutoff value of 8. Approximately 232,000 reads were produced.

### Sequence analyses

Maximum-likelihood trees for each mosquito-associated orbivirus segment of the PHSV clade were computed from aa and coding sequences that were downloaded from NCBI Virus (Table S5) [33]. NCBI Open Reading Frame Finder [34] was used separately to determine coding sequences of MBV Ilomantsi strain and KEVOOV segments. The sequences were first aligned using MAFFT v7.490 [35], and then, the maximum-likelihood trees were built with IQ-TREE v2.1.4-beta [36] using ultrafast bootstrap approximation and ModelFinder for selecting the most optimal substitution model (general matrix LG for aa trees and general time reversible model for nt trees) [37,38]. Each tree was rooted midpoint with the BTV (GenBank accession numbers: PQ303740–PQ303749), AHSV-1 (OL581614–OL581623), EHDV-1 (NC_013396–NC_013405), Wallal virus (KJ495745–KJ495754), Eubenangee virus (NC_038592- NC_038593) and Orungo virus (NC_038604–NC_038613.1). The resulting trees were visualized and rooted in R statistical computing platform (v4.4.2) using the following packages: ggplot2 (v3.5.1), ggtree (v3.14.0), treeio (v1.30.0), tibble (v3.2.1), readr (v2.1.5), ape (v5.8), dplyr (v1.1.4), phangorn (2.12.1) and patchwork (v1.3.0).

Additional characterization of the orbivirus genomes included verifying encoded viral proteins using NCBI Conserved Domain Search and NCBI blastx [39,41], determining GC content and genome size with Seqkit (v2.3.0) [42], calculating sequence identities among genomic segments using EMBL-EBI Clustal Omega [43] and identifying possible recombination events with Recombination Detection Program (v5.57) (Table S5) [44].

### PAGE of viral RNA

Total RNA was extracted from infected C6/36 cells using TRIzol reagent (Thermo Fisher Scientific) with added glycogen according to the manufacturer’s instructions and dissolved in PCR grade H_2_O. Ten per cent polyacrylamide (PAGE) gels were prepared consisting of 1.5 M Tris-HCl (pH 8.8), 30% acrylamide solution (acrylamide/bisacrylamide, Sigma-Aldrich), tetramethylethylenediamine (TEMED) (Promega), 10% ammonium persulphate, Milli-Q water and 0.5 M Tris-HCl (pH 6.8). Tris-glycine (pH 8.3) buffer was used as a running buffer. RNA samples were mixed with an equal volume of DNA loading buffer and run for ~3 h at 180 V at RT. After electrophoresis, the gel was stained with ethidium bromide for 15 min and imaged using FAS-Digi Pro (Nippon Genetics).

### Transmission electron microscopy

C6/36 cells infected with KEVOOV were harvested 5 days post-infection and fixed in 5% paraformaldehyde and 5% glutaraldehyde in 0.1 M Na-cacodylate (NaCac) for 3 h at RT, scraped into 2 ml of refrigerated 0.1 M NaCac and then centrifuged at 1,200 r.p.m. for 10 min at 4 °C. The supernatant was discarded, and cells were resuspended in 4 ml of refrigerated 0.1 M NaCac before being transferred to a new Eppendorf tube and centrifuged at 1,200 r.p.m. for 10 min at 4 °C. Once more, the supernatant was discarded, and 500 µl of refrigerated 0.1 M NaCac was used to cover the remaining cells. The sample was then transferred to the Electron Microscopy Unit of the Institute of Biotechnology, University of Helsinki, for further processing and post-staining with uranyl acetate and lead citrate [45]. Imaging was done on Jeol JEM-1400 Transmission Electron Microscope (Jeol Ltd., Tokyo, Japan) at 80,000 V. The transmission electron microscopy (TEM) images were analysed by using Microscopy Image Browser (MIB) v2.9020 (https://mib.helsinki.fi/index.html) [46].

### Cell line testing

Confluent BHK-21 T25 cm^2^ (baby hamster kidney, ATCC CCL-10) and SCP T25 cm^2^ (sheep chorion plexus, obtained from the cell line collection of the Department of Virology, University of Helsinki) cells were infected with 100 µl of KEVOOV and MBV Ilomantsi isolates (passage 1 from C6/36 cells) as described before. After infections, MEM with 2% FBS and l-glutamine was added onto cells, and cells were kept at 37 °C, 5% CO_2_. In the absence of CPE, the infected BHK-21 cells were passaged four times and observed for CPE for a total duration of 29 days. SCP cells were passaged once and observed for 20 days for CPE. From the final passage, RNA was extracted from infected cells as previously described with TRIzol, and RNA PAGE and/or sequencing was performed as previously described for viral genome detection.

## Results

### Identification of orbivirus sequences from virus isolation next-generation sequencing data

In this study, virus isolation trials were conducted from 94 species-specific pools of 9 *Ochlerotatus* species previously collected in Finland and studied for their RNA viromes [26]. The virus isolation trials were performed in mosquito C6/36 and mammalian Vero E6 cells, since they are well documented to support the replication of a wide range of viruses. Various levels of CPEs were observed in several of the C6/36 virus isolations, whereas no clear CPE was observed in the Vero E6 cells. To detect RNA viruses, RNA extracted from the supernatant samples of all virus isolation cultures was processed for Illumina MiSeq sequencing in pools. Viral sequences were identified from the next-generation sequencing (NGS) data using the Lazypipe virus discovery, assembly and taxonomic profiling pipeline [30]. Most of the viral reads were identified as insect viruses (data not shown), but two of the pooled sequencing samples from infected C6/36 cells also contained animal orbivirus reads. As the focus of the study was arthropod-borne viruses, and not insect viruses, the following work concentrated on the pools containing animal orbivirus reads. The original virus isolation supernatant samples comprising the animal orbivirus-positive pooled sequencing samples were then individually passaged and sequenced. The confirmatory sequencing identified two separate orbivirus isolates from C6/36 cultures, from samples #70 and #88. Based on Lazypipe results of the Illumina MiSeq sequence data [30], the supernatants of samples #70 and #88 yielded 560,032 reads in total. Sample #70 yielded 66,237 viral reads (11.8%), most of them being assigned to orbiviruses (*n*=58,191, 87.9%), namely, MBV. The sample also contained reads related to Dezidougou and Cordoba negeviruses (data not shown), suggesting co-isolation of insect negeviruses. In total, 314,853 reads were obtained from sample #88, of which 261,842 were assigned to orbiviruses (83.2%) and included CHeRI orbivirus-related reads (*n*=259,489, or 99.1%) and GXOV-related reads (*n*=2,353, 0.9%). No other viral sequences were detected from sample #88, suggesting that a single orbivirus strain was isolated from this sample.

Orbivirus sequences from mosquito pools #70 and #88 were detected only from C6/36 cells and not from the corresponding Vero E6 cells that had been infected with the same mosquito homogenate samples. During the two passages, sample #70 caused CPE on C6/36 cells 12 days and 6 days post-infection, respectively. This faster CPE in the second passage could be partially due to negeviruses being present in the sample. Similarly for sample #88, mild CPE was visible on days 14 and 10 post-infection in the first and second passages, respectively. The CPE on the C6/36 cells in both virus samples was modest and similar: small, rounded cells or cell clumps were observed to detach from the cell monolayer. Additional mammalian cell line infection experiments were performed using virus stocks collected from infected C6/36 cells of orbivirus isolates #70 or #88 on BHK-21 and SCP cells. These did not yield CPE, and no viral RNA was detected from infected cells in RNA PAGE or by sequencing.

### Virus genome characterization and phylogenetic analysis

#### Isolate #70

**Fig. 2.**
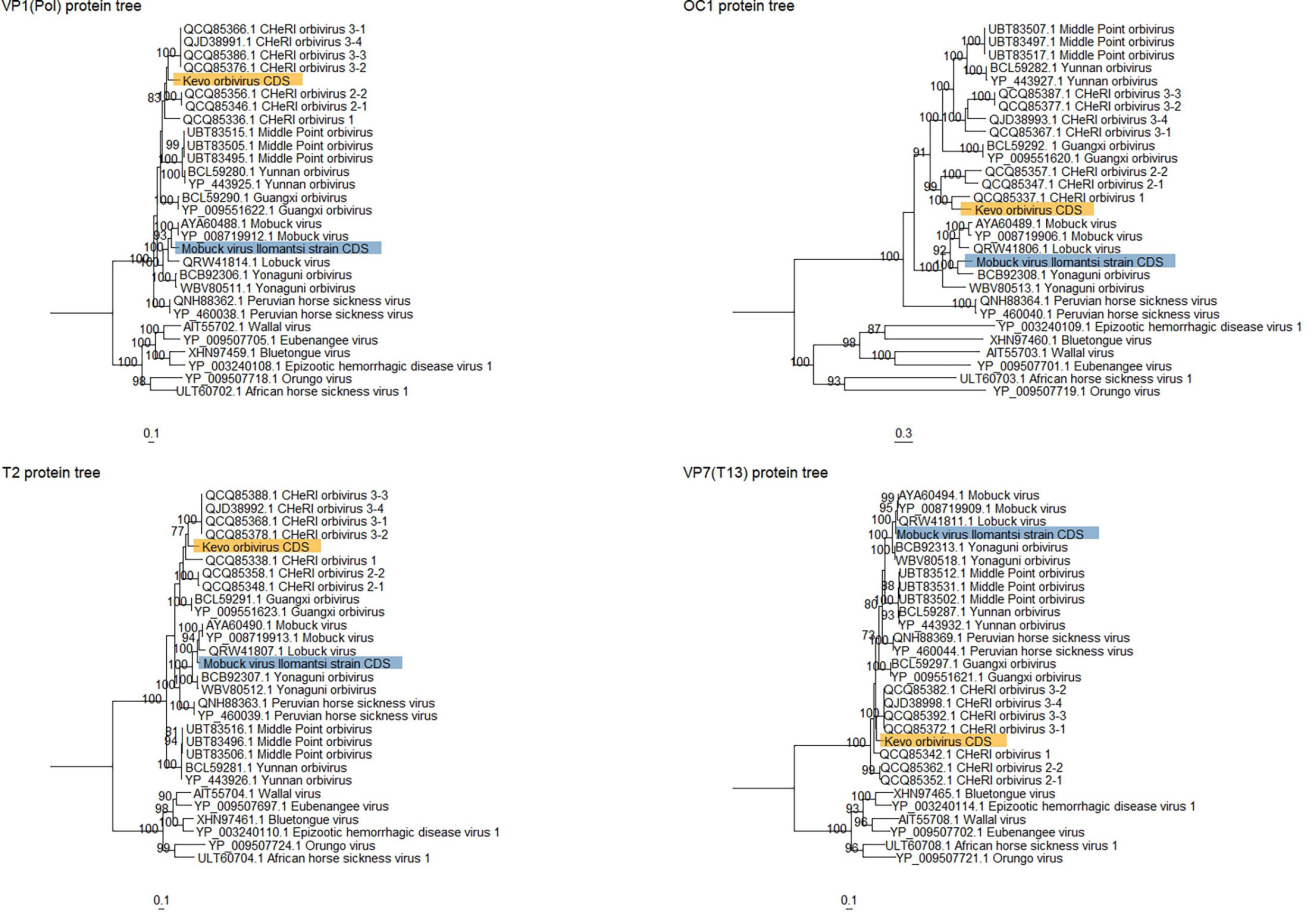
Maximum-likelihood trees based on ^aa^ sequences of KEVOOV and MBV Ilomantsi strains VP1, OC1, T2 and VP7(T13). The outgroups were EHDV-1, BTV, AHSV-1, Wallal virus (WALV), Eubenangee virus (EUBV) and Orungo virus (ORUV). Bootstrap values over 70 are shown. KEVOOV is highlighted in orange and the MBV Ilomantsi strain in blue.

**Fig. 3.**
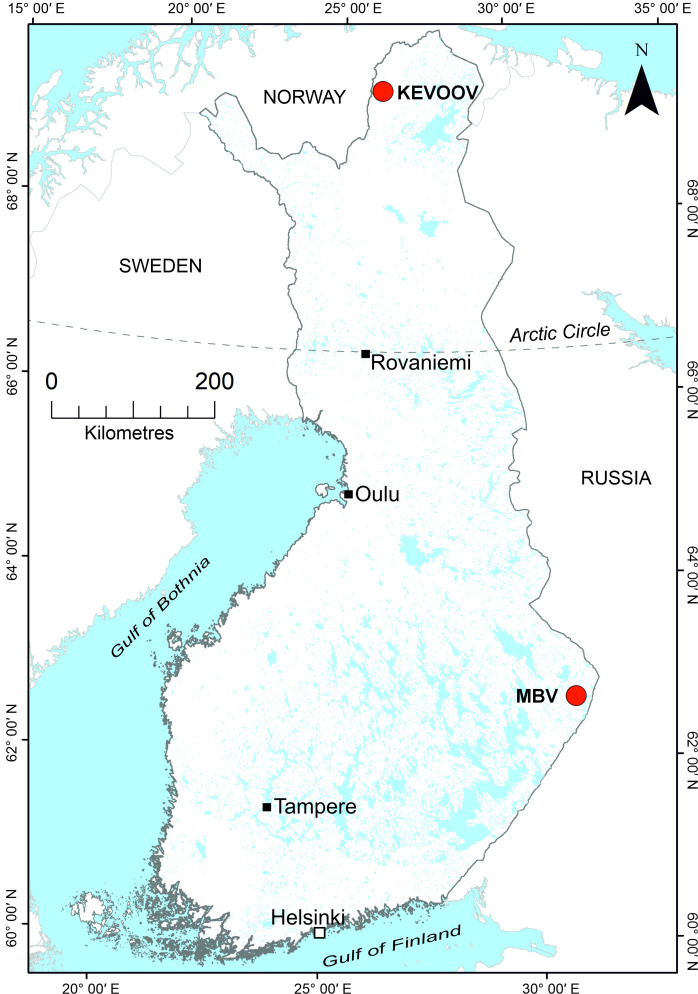
Map of Finland showing the collection locations of the virus-positive mosquito pools MBV Ilomantsi strain and KEVOOV. The map was created using ESRI ArcGIS v10.3.1 (ESRI, Redlands, CA, USA).

Phylogenetic analyses were conducted with aa (Figs 2, S1 and S2) and nt (Figs S3–S5) sequences separately for each of the gene segments and their encoded proteins. The phylogenetic analysis of the viral genes uniformly suggested that isolate #70 is most closely related to MBV. A near complete, 20,373 nt, genomic sequence of isolate #70 (Table 1) was obtained, which was similar to previous MBV isolates [19,20]. It comprised 10 segments that encoded 10 proteins, with segments varying from 831 nt (segment 10) to 4,091 nt (segment 1) in length. Additionally, we detected putative ORFs with ORFfinder for genes encoding NS4 and NS5 in segments 9 and 10, respectively. The former was 303 nt long and located in the nt positions 290–592, and the latter was 93 nt long located in the positions 215–307. Although these are similar to other orbiviruses that have said genes in size and position in the segments, they were highly divergent in nt compositions. Therefore, whether these ORFs encode actual proteins remains unverified until further protein detections and/or studies. The GC content varied between 34.1% and 42.7%, averaging at 36.5% (Table 1). Analyses of isolate #70 using the RDP5 program [44] did not detect recombination or reassortments in the genomic sequence, but the sequence had over 78% identity in VP1 aa with LBV and other MBV strains. When comparing aa and nt sequences of VP3(T2), isolate #70 shared identities of over 83% and 76%, respectively, with two other MBV strains, LBV and YONOVs (Tables S1–S3), therefore suggesting that isolate #70 should be considered as a divergent strain of MBV rather than a new species. Based on the collection site, in Ilomantsi, North Karelia, in Eastern Finland (62° 43.131′ N 31° 00.301′ E), the strain was designated as an MBV Ilomantsi strain (Fig. 3). The genome sequences have been deposited to GenBank with accession numbers PQ165799–PQ165806, PQ505671 and PQ505672 (Table 1). MBV Ilomantsi strain was isolated from a pool of 20 female *Ochlerotatus communis* collected in late June 2015.

**Table 1.** Genome details and GenBank accession numbers of KEVOOV and MBV Ilomantsi strain. Partial sequences are marked with *. MBV Ilomantsi strain lacks 13 nt from the segment 6 VP5 5′ end. It is to be noted that because MBV Ilomantsi strain has partial sequences, the segment numbering can change when more complete sequence is obtained

#### Isolate #88

Phylogenetic analyses were conducted for aa (Figs 2, S1 and S2) and nt (Figs S3–S5) sequences separately for each of the ten segments of isolate #88. The resulting trees uniformly suggested that isolate #88 was divergent from isolate #70 and most closely related to different CHeRI orbiviruses. The exact position varied from an outgroup to a position within the group (Figs 2 and S1–S5). The complete genome of isolate #88 was 19,843 nt, which is in line with the genome sizes of other mosquito-associated orbiviruses. Its ten segments vary from 816 nt (segment 10) to 3,983 nt (segment 1) in length. The GC content varied between 33.3% and 40.4% averaging at 35.9% (Table 1). The genome encoded 11 proteins, including segment 9 which encoded VP6 and also NS4 (Table 1). Furthermore, segment 10 was a putative, yet unverified ORF for NS5 (93 nt long in nt positions 188–280). As the obtained isolate #88 sequence was divergent from those in GenBank, and the 5′- and 3′- untranslated, non-coding regions (NCRs) located at the segment termini were not completely obtained in direct RNA sequencing, these were amplified and sequenced separately from concentrated virus extracted RNA for obtaining a complete genome sequence. The segment 5′- NCRs were 7–29 nt long; however, in segment 7 (which encodes NS2), the 5′ NCR length was 73 nt (Table 1). The NCR in the 3′-ends varied between 3 and 104 nt, and longer NCRs were observed in segment 5 (encodes NS1), which was 195 nt, and in segment 7 (encodes NS2), it was 184 nt (Table 1). Similar longer NCRs in segments 5 and 7 have been observed in MBV. We then determined the conserved termini for each segment (Table 1). The consensus sequences for termini were 5′-GUUAAAA and (_G/U_)(_U/C/A_)(_G/U/C/A_)(_A/U/C_)(_A/G/U_)(_G/A/C_)(_A/G/U_)UAC-3′. The conserved 5′-termini of isolate #88 were most similar to PHSV (5′-GUUAAAA) [3], and the conserved UAC-′3 region in the 3′ termini was similar to other orbiviruses [3]. No recombination or reassortment was detected using RDP5 in the genomic sequence of isolate #88.

Based on the International Committee on Taxonomy of Viruses (ICTV) species demarcation criteria, isolate #88 fulfils the criteria to be considered a novel orbivirus species. It states that viruses belonging to the same species have over 76% nt identity and over 83% aa identity in VP3(T2), while those in different species usually have below 74% nt identity. The closest relatives to KEVOOV, based on the VP2(T2), are CHeRI orbiviruses 3.1–3.4 with nt identities ranging from 70.9% (CHeRI orbivirus 3–1) to 71.4% (CHeRI orbivirus 3–2) (Table S1) and aa identities of 74.8% (Table S2). Furthermore, according to ICTV, members of the same species also have over 78% aa identity in VP1, which encodes the viral RNA-dependent RNA polymerase (RdRP). Isolate #88 has 75.9% aa identity for VP1 when compared with the CHeRI orbivirus 3–4 (Table S3). Thus, based on the identities and the phylogenetic analyses, we propose that isolate #88 should be considered as a novel orbivirus species. We designated this virus as KEVOOV after the site where the 21 female *Oc. communis* comprising the virus-positive pool were collected in late July in 2015, near the Kevo nature reserve in Utsjoki (69° 25.069′ N 26° 10.852′ E), in northern Finland (Fig. 3). The KEVOOV genomic data has been deposited to GenBank with the following accession numbers: PQ165789–PQ165798.

### RNA PAGE

**Fig. 4.**
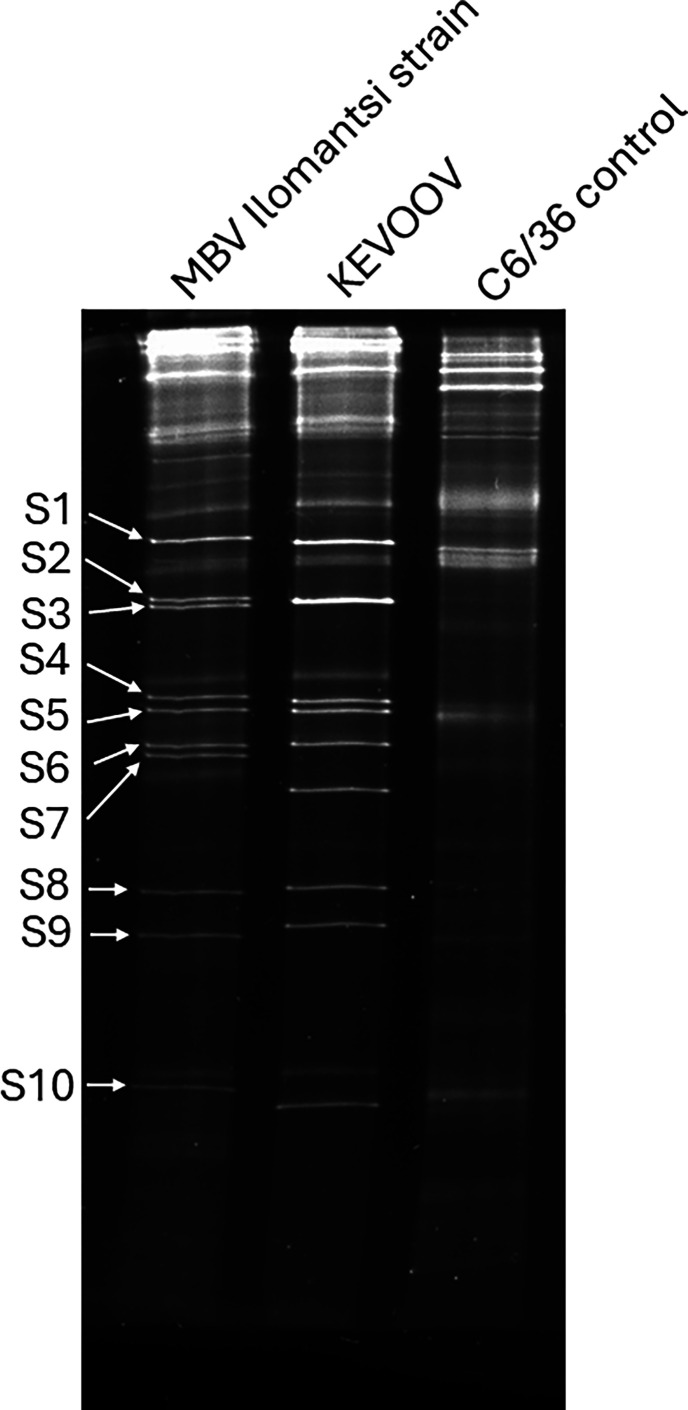
Migration patterns of genomic RNA of KEVOOV and the MBV Ilomantsi strain in PAGE. Segments are labelled S1–S10 from largest to the smallest. An RNA sample from uninfected C6/36 cells was included as a control.

The total RNA extracted from KEVOOV and MBV Ilomantsi strain-infected C6/36 cells yielded visible ten genomic segments in PAGE that were not seen in the clean cell control and that were similar to those reported for other orbiviruses. The migration patterns of both viruses differ from those of *Culicoides*-borne and tick-borne viruses but most closely resembled the pattern of the mosquito-associated GXOV [25] as well as the mosquito-borne YUOV and PHSV [10, 12, 47]. Notably, the migration pattern of KEVOOV and the MBV Ilomantsi strain differed in segments 2, 3, 6 and 7 (Fig. 4). While KEVOOV segments 2 and 3 appeared to co-migrate, those of MBV Ilomantsi migrated separately. With segments 6 and 7, the MBV Ilomantsi strain segments migrated closely together compared with KEVOOV (Fig. 4).

#### TEM of KEVOOV

**Fig. 5.**
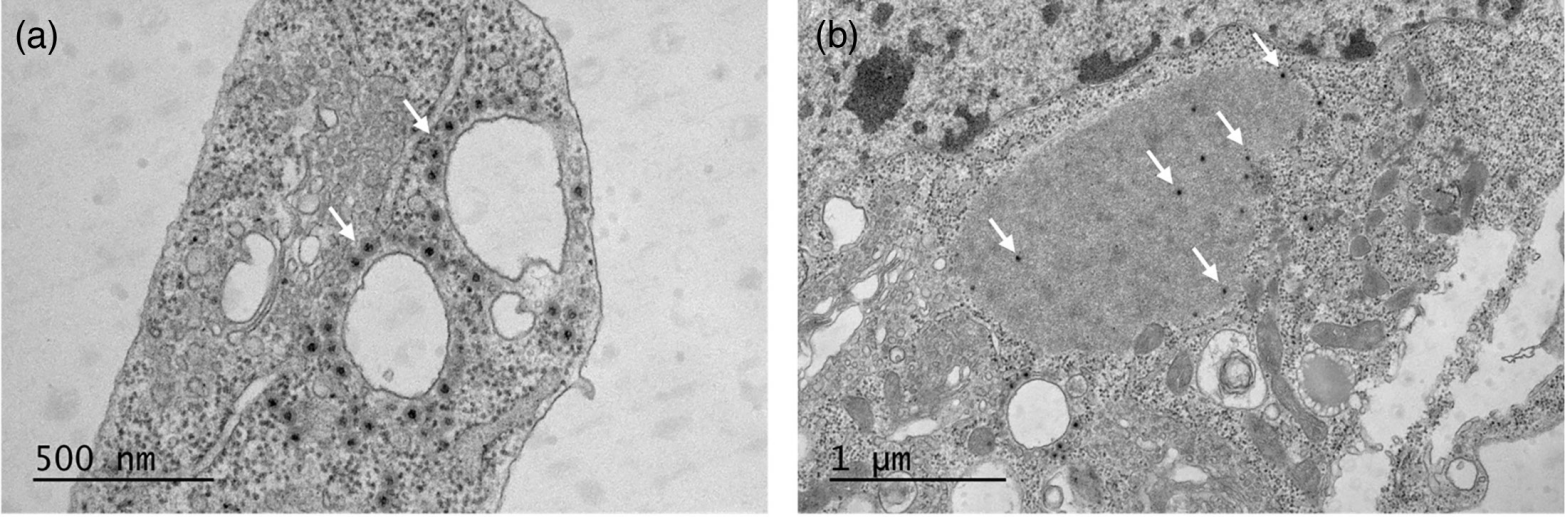
Transmission electron microscopic image of C6/36 cells infected with KEVOOV. White arrows indicate KEVOOV particles. (a) KEVOOV particles surrounding the vacuoles. (b) KEVOOV particles in the cell cytoplasm.

Spherical particles consistent with the morphology of orbivirus virions were visible in KEVOOV-infected C6/36 cells in TEM (Fig. 5a, b). Particles of resembling orbivirus virions [4] were located in the cell cytoplasm (Fig. 5b). Particle sizes varied between 50 and 66 nm. These sizes correspond to the previously described BTV pre-subcore (~55 nm) and core (~68 nm) sizes and CHeRI orbivirus 1 [3,4, 48]. Several individual KEVOOV particles were also detected around these vacuole-like structures (Fig. 5a, b).

## Discussion

Currently, little is known about the orbivirus diversity in Finland. Prior to this study describing orbiviruses from Finnish mosquitoes, only serological evidence had been reported in Finland in the 1970s from cattle against a tick-borne orbivirus, namely, the Kemerovo group Tribeč virus [49]. Thus far, the major orbiviral animal pathogens, such as the *Culicoides-*transmitted EHDV, AHSV and BTV, have not been detected in Finland [50,53]. As BTV has been spreading northwards with recent outbreaks in Sweden, Denmark and Norway [54,56], annual surveillance of bovine blood samples is performed also in Finland for BTV RNA and antibodies [57], but continuous surveillance of orbiviruses in vectors is currently not performed.

By utilizing a methodology combining cell culture virus isolation, NGS and automated bioinformatic virus identification tool, we were able to discover for the first time two vertebrate orbiviruses from Finnish mosquitoes. These were not initially detected in the direct sequencing of mosquito homogenate RNA [26], possibly due to the stringent selection of sequences for the virome analysis, such as high detection limit, minimum contig length of over 1,000 nt and similarity to viral RdRP sequences. There may have been low amounts of viral RNA in the sequencing samples, and virus isolation success from such samples was likely influenced by the stability of infectiousness of the orbivirus virions [3] and the use of a mosquito cell line apparently highly susceptible to mosquito-associated orbiviruses.

The two orbiviruses isolated in this study from *Oc. communis* mosquitoes were distinct, but related. Based on the complete genome sequence of KEVOOV and a nearly complete genome of MBV strain Ilomantsi, these isolates have genetic characteristics typical for mosquito-associated orbiviruses including their genomic segment size, GC content and phylogenetic position. KEVOOV segment 9 was found to encode an additional protein NS4. In studies performed on other orbiviruses, this protein has been found to be multifunctional and to have a role as a virulence factor involved in modulating cellular innate responses in mammalian cells [58]. Currently, there is no information on the relevance of NS4 protein for mosquito-borne orbivirus pathogenesis, although apparently, e.g. YUOV [13] and PHSV [59] also encode for this protein [58].

Based on the ICTV criteria, KEVOOV originating from the Arctic is a potentially new species of orbivirus, whereas the isolate from Ilomantsi is proposed to be taxonomically a strain of MBV. KEVOOV and MBV Ilomantsi are phylogenetically positioned within the PHSV group, which includes many viruses lacking detailed characterization of vectors and disease associations and which was originally described as side findings of surveillance studies targeting other viruses [15,21,23] or from sick animals [4,10, 19]. One property of the PHSV group viruses is their preference for mosquito cells for isolation and *in vitro* culture. This was also the case for KEVOOV and MBV Ilomantsi strain that were isolated only in C6/36 but not in Vero E6 cells. Although the PHSV group viruses are animal viruses that can infect various mammalian species, their ability to grow in mammalian cells *in vitro* varies. The observed inability of KEVOOV and MBV Ilomantsi to infect the tested mammalian (hamster, sheep and monkey) cells is not atypical for PHSV group viruses. Several cell lines including human [12,14], rodent [10,12, 22] and primate [4,12, 13, 15, 16] origin have been tested for several PHSV group viruses, but only some viruses (MPOV, PHSV, ELSV and YONOV strain JH2019C603) have been shown to replicate in cultured mammalian cells.

As mosquito-borne and mosquito-associated orbiviruses are geographically widespread, it was not completely unexpected to discover them from mosquitoes in northern Europe. To the best of our knowledge, this is the first report of mosquito-associated orbiviruses isolated in Finland or elsewhere in northern Europe. This is also the first time MBV has been isolated outside of the USA and isolated from mosquitoes, also associating the virus with vector species *Oc. communis*. Notably, *Oc. communis* is a native and widespread species in Finland [60], which aggressively seeks bloodmeals and prefers to feed upon mammals [61]. The phylogenetically most closely related viruses for KEVOOV and MBV Ilomantsi strain, namely, the CHeRI orbiviruses and MBV, are described from sick or deceased white-tailed deer (*Odocoileus virginianus*) in the USA [4,19]. These viruses have been mostly, but not exclusively, detected from co-infections with a *Culicoides*-borne orbivirus EHDV [4,18]. The significance of the reported co-infections in the deer disease is not defined, leaving the pathogenic properties of CHeRI orbiviruses and MBVs unclear. Notably, infections without EHDV co-infection have also been described for a few CHeRI strains, namely, the CHeRI orbivirus 1 and 3-3 in white-tailed deer. The white-tailed deer are endemic wild animals in Finland; however, those are apparently not common in the areas of origin of the Finnish orbivirus isolates. MBV Ilomantsi strain was found from mosquitoes collected in Eastern Finland, where white-tailed deer appear to be absent, but other ruminants such as elk (*Alces alces*) [62] are present. White-tailed deer are also absent in the Arctic, where KEVOOV was discovered. Instead, reindeer (*Rangifer tarandus*) are very common. Further studies are required to elucidate the distribution and possible animal disease associations of KEVOOV and the MBV Ilomantsi strain in Finland and beyond in northern Europe.

## Concluding statements

This study contributes to the understanding of the diversity and global distribution of mosquito-associated orbiviruses that extend to northern Europe. We conclude that KEVOOV and MBV Ilomantsi represent possibly novel vector-borne animal pathogens for Finland.

## Abbreviations

AHSV: African horse sickness virus
BTV: bluetongue virus
CHeRI: Cervidae Health Research Initiative
CPE: cytopathic effect
EHDV: epizootic haemorrhagic disease virus
ELSV: Elsey virus
EUBV: Eubenangee virus
GXOV: Guangxi orbivirus
ICTV: International Committee on Taxonomy of Viruses
KEVOOV: kevo orbivirus
LBV: lobuck virus
MBV: mobuck virus
MEM: minimum essential medium
MPOV: middle point orbivirus
NCR: non-coding region
NGS: next-generation sequencing
ONT: Oxford Nanopore Technologies
ORF: open reading frame
ORUV: Orungo virus
PHSV: peruvian horse sickness virus
RdRP: RNA-dependent RNA polymerase
RT: room temperature
TEM: transmission electron microscopy
TEMED: tetramethylethylenediamine
WALV: Wallal virus
YONOV: yonaguni orbivirus
YOUV: yunnan orbivirus
